# Assessment of Esophageal High-Resolution Impedance Manometry in Patients with Nonobstructive Dysphagia

**DOI:** 10.1155/2018/6272515

**Published:** 2018-05-02

**Authors:** Zhaoyu Liu, Jiazhi Liao, Dean Tian, Mei Liu, Zili Dan, Qin Yu

**Affiliations:** Department of Gastroenterology & Hepatology, Tongji Hospital, Tongji Medical College, Huazhong University of Science and Technology, Wuhan 430030, China

## Abstract

**Background:**

High-resolution impedance manometry (HRIM) can calculate the bolus motion parameters and the ratio of complete esophageal transit besides the conventional esophageal dynamic parameters; therefore, we could better manage the patients with nonobstructive dysphagia (NOD) clinically.

**Aim:**

To analyze the HRIM parameter results of NOD patients and evaluate the characteristics of their esophageal motility and transit function.

**Methods:**

In total, 58 NOD patients were assessed and the clinical diagnoses were determined. HRIM was performed, and both conventional high-resolution manometry and esophageal transit parameters were analyzed.

**Results:**

In 58 NOD patients, 28 patients had achalasia, 3 esophagogastric junction outflow obstruction, and 20 nonspecific esophageal motility disorders, and 7 were normal. Impedance results demonstrated that all the patients with achalasia exhibited incomplete esophageal transit (ICET), three patients with esophagogastric junction outflow obstruction showed ICET, and the average bolus transit time (BTT) was 6.6 ± 1.2 sec. In 20 nonspecific esophageal motility disorders, 13 patients with gastroenterologly reflux disease (GERD) presented ineffective esophageal motility and fragmented peristalsis, and 65.0% swallows had exhibited ICET. However, 49.1% swallows of 7 nonspecific esophageal motility disorder patients with non-GERD had exhibited ICET. The average BTT in 13 GERD patients was longer than that in the non-GERD patients (8.1 ± 1.1 sec versus 5.5 ± 0.3 sec, *P* < 0.05). And in the seven patients with normal esophagus function, 3.5% swallows showed ICET and BTT was 5.6 ± 0.3 sec.

**Conclusion:**

Achalasia was the most common esophageal dysmotility in NOD patients, followed by nonspecific esophageal motility disorders. The clinical diagnoses of NOD were mostly achalasia and GERD. Impedance assessments showed that all achalasia cases exhibited ICET, and other esophageal motility abnormalities that represented ICET were associated with contraction break and ineffective swallow. Compared to non-GERD patients, BTT was significantly prolonged in patients with GERD.

## 1. Introduction

Esophageal high-resolution manometry (HRM) is the current state-of-the-art diagnostic tool to evaluate esophageal motility patterns and is widely used in clinical practice [1]. It has already proved to be the standard diagnostic method taking over conventional water perfused manometry in the assessment of nonobstructive esophageal motility disorders. In addition, HRM is a very helpful method to understand distinct properties of esophageal motility; therefore, it could better characterize the mechanisms of gastroesophageal reflux disease (GERD) and abnormal esophageal body motion [2]. However, HRM cannot provide information about bolus transport whose conclusions have been based on studies combining manometry with radiological visualization of flow and clearance [3].

More recently, high-resolution impedance manometry (HRIM) has been introduced, and this technique is very similar to conventional manometry system, whereas impedance should be regarded as an add-on, which combines the benefits of HRM and impedance-based bolus transit assessments. Multichannel intraluminal electrical impedance (MII) measurement has provided a sensitive means of evaluating bolus movement and esophageal clearance without radiation. The principles of impedance technique are based on measurement of electrical impedance differences in resistance to current of the intraluminal contents. Using different substances having different impedances, MII could distinguish the intraluminal air which exhibits high impedance from liquid which exhibits low impedance. Validation studies have verified that MII measurement has high sensitivity and accuracy for detecting intraesophageal bolus movement and monitoring reflux [4, 5]. Therefore, the aim of this study was to evaluate the characteristics of nonobstructive dysphagia (NOD) through investigating the data of HRIM in these patients, in order to better understand the pathophysiologic mechanisms of NOD.

## 2. Materials and Methods

### 2.1. Subjects

Patients who suffered from nonesophageal or obstructive dysphagia were excluded through upper gastrointestinal endoscopy and barium radiography examination, and finally a total of 58 NOD patients (32 men, 26 women, mean age 47 years; range 22–80) from Aug. 2016 to Dec. 2016 at Tongji Hospital, Tongji Medical College, Huazhong University of Science and Technology, were enrolled in our study.

### 2.2. Study Protocol

All NOD patients discontinued all medications that might affect gastrointestinal motility 3 days before examination. A combination of high-resolution solid-state manometry and impedance study was done in each subject after a 6-hour fast. The HRIM catheter was a 4.2 mm outer diameter solid-state with 36 circumferential pressure sensors at 1 cm interval. Impedance measuring segments included 18 segments at 2 cm intervals (ManoscanTM, Sierra Scientific Instruments Inc.). The HRIM assembly was calibrated at 0 and 300 mmHg using externally applied pressure prior to the study. Then, the catheter was placed transnasally and positioned to record from the hypopharynx to the stomach with approximately five intragastric pressure sensors. The HRIM protocol is as follows: firstly, a 5 min baseline recording, then ten 5 ml swallows of normal saline in a supine position for test swallows at 20–30 s intervals. Normal saline was used instead of regular water since it has a standardized ionic concentration and provides better impedance changes.

### 2.3. Esophageal Manometry Characteristic Interpretation

Pressure topography was analyzed manually using ManoViewTM software with data tracings viewed in the color pressure topography mode. The integrated relaxation pressure (IRP) which is the mean of 4 s of maximal deglutitive relaxation in the 10 s window beginning at upper esophageal sphincter (UES) relaxation [6] was used to evaluate esophagogastric junction (EGJ) relaxation. In total, the lengths of lower esophageal sphincter (LESL), midrespiratory resting pressure (LESP), and IRP were applied to assess LES function. UES pressure and UES residual pressure during swallowing were applied to assess UES function.

The distal contractile integral (DCI) which integrates the length (centimeter), contractile pressure (mmHg), and duration (second) of contraction at 20 mmHg of isobaric contour reflects the magnitude of the distal esophageal contraction. It was proposed to incorporate the LES into the DCI measurement domain. Failed and weak contractions were defined as DCI < 100 mmHg·s·cm and >100 mmHg·s·cm but <450 mmHg·s·cm, respectively [6]. The contractile deceleration point (CDP), defined as the point where esophagus propagation decelerates in velocity, marked a transition from esophageal peristaltic clearance to emptying of the phrenic ampulla, thus provided a reliable landmark for measuring peristaltic velocity. Distal latency (DL) was measured as the interval from upper esophageal sphincter relaxation to the CDP [7]; a value less than 4.5 s defined a premature contraction. The definition of distal esophageal spasm (DES) depended on ≥20% premature contractions and normal mean IRP [6]. Hiatal hernia was defined using the criterion of separation between the LES and crural diaphragm (CD) during the baseline recording [8]. Large break was defined as >5 cm in the 20 mmHg isobaric contour, which was significantly more common in patients with dysphagia [9].

### 2.4. Esophageal Manometry Impedance Interpretation

In conjunction with HRM, impedance monitoring allowed tracking the swallowed bolus in relation to esophageal pressure topography. Bolus transit time (BTT) defined as time elapsed between bolus entry at 19 cm above the reference line and bolus exit at 5 cm above the reference line [10]. Swallows can then be classified as having complete esophageal transit (CET) if bolus entry was seen at the most proximal site, and bolus exit was recorded in all distal impedance measuring sites, or incomplete esophageal transit (ICET), if bolus exit was not identified at one or more of the distal impedance measuring sites [11]. For an individual patient, abnormal bolus transit was defined as ≥30% liquid swallows with ICET [12].

### 2.5. Clinical Diagnostic Criteria for Achalasia and GERD

Achalasia is a rare neurodegenerative motility disorder that is characterized by loss of peristalsis and failure of relaxation of the LES, especially during swallowing [13, 14]. The diagnostic criterion for achalasia is at least consistent with the following items: barium esophagogram may reveal a classic “bird's break” appearance, esophageal dilation, or a corkscrew appearance with aperistalsis; HRM manifests aperistalsis and failure of relaxation of the LES [15, 16]. The diagnosis of GERD requires any of the following besides presence of persistent symptoms like heartburn and reflux suggestive of GERD: presence of erosive esophagitis and 24 h pH impedance exhibited pathological esophageal acid exposure and/or symptom-reflux association [17].

### 2.6. Ethical Considerations

The study protocol was approved by the Ethics Committee of Tongji Hospital, Tongji Medical College, Huazhong University of Science and Technology.

### 2.7. Statistical Analysis

The HRIM descriptive statistics for all continuous and ordinal measures were presented as medians with interquartile ranges (IOQ). ANOVA tests were utilized to compare mean (or median) values of continuous outcomes across classification types. LSD test was used to compare between groups. Spearman's rank correlation test was used to identify correlation between bolus clearance and the length of breaks for the 30 mmHg isobaric contours. Analyses assumed a 5% level of statistical significance, and all statistical analyses were performed using SPSS version 19.0 (SPSS Inc., Chicago, USA).

## 3. Results

### 3.1. Characteristics of Patients

**Figure 1:**
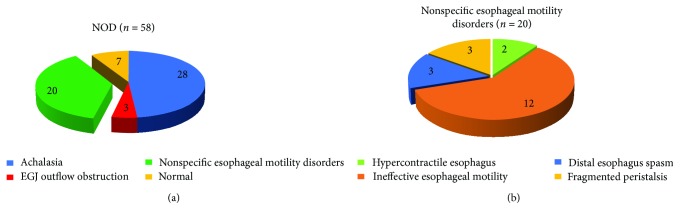
Characteristics of nonobstructive dysphagia (NOD) patients. (a) In a total of 58 patients, 28 (48.3%) patients were diagnosed with achalasia, 3 (5.2%) patients with EGJ outflow obstruction, and 20 (34.5%) with nonspecific esophageal motility disorders, and 7 (12.1%) patients were normal. (b) Of 20 nonspecific esophageal motility disorders patients, 3 patients had fragmented peristalsis, 3 patients had distal esophageal spasm, 2 patients had hypercontractile esophagus, and 12 patients had ineffective esophageal motility.

A total of 58 NOD patients (32 men, 26 women, mean age 47 years; range 22–80) were enrolled in this study. 28 (48.3%) patients were diagnosed with achalasia, of which 9 cases were I type achalasia, 18 cases were II type achalasia, and 1 case was III type achalasia. Three (5.2%) patients had EGJ outflow obstruction, 3 (5.2%) patients had distal esophageal spasm, 2 (3.4%) patients had hypercontractile esophagus, 3 (5.2%) patients had fragmented peristalsis, and 12 (17.2%) had ineffective esophageal motility (IEM), which are defined as ≥50% infective swallows and DCI < 450 mmHg·s·cm [6]. And 7 (12.1%) patients were normal (Figure 1).

Of the 58 patients of NOD, 13 (22.4%) patients were diagnosed with GERD in clinic, including 5 cases with hiatal hernia, 1 with hypercontractile esophagus, and 7 with ineffective swallows.

### 3.2. Evaluation of Esophageal Dynamic Characteristics

Out of the 58 patients with NOD, LESP and 4sIRP were significantly higher in achalasia and EGJ outflow obstruction patients, and there were no significant differences among the length of LES, UESP, and UES residue pressure (Table 1).

All 28 achalasia patients presented aperistalsis, and 19 (67.9%) patients showed synchronous contractions and panesophageal pressurization. Of the 3 EGJ outflow obstruction patients, 1 patient showed large break and ineffective swallow, and 2 patients exhibited synchronous contractions. The mean pressure of esophageal body was 55.1 ± 7.2 mmHg, and DCI was 1400.5 ± 428.2 mmHg·s·cm. In 20 patients of nonspecific esophageal motility disorder (NEMD), 13 patients exhibited GERD and the other 7 patients did not. Compared to non-GERD patients, the NEMD patients with GERD showed an obvious lower mean esophageal body pressure (49.8 ± 5.8 mmHg) and DCI (699.1 ± 123.1 mmHg·s·cm) (*P* < 0.05) (Table 2). It is worth mentioning that the NEMD patients without GERD also exhibited a significant lower mean body pressure (75.8 ± 12.5 mmHg versus 128.4 ± 4.2 mmHg) and DCI (1258.3 ± 206.8 mmHg·s·cm versus 2344.6 ± 406.6 mmHg·s·cm) compared to normal esophagus function patients (*P* < 0.05) (Table 2).

### 3.3. Evaluation of Bolus Transit Using HRIM

In total, 580 swallows were recorded. All achalasia patients showed ICET, regardless of their type. Of the three patients with EGJ outflow obstruction, only three swallows showed CET, and the other 27 swallows showed ICET. The average BTT was 6.6 ± 1.2 sec. In 13 NEMD patients with GERD, 65.0% swallows exhibited ICET, while 49.1% swallows of 7 NEMD patients with non-GERD exhibited ICET. The average BTT in 13 GERD patients was longer than that in non-GERD patients (8.1 ± 1.1 sec versus 5.5 ± 0.3 sec, *P* < 0.05). And in the seven patients with normal esophagus function, 3.5% swallows showed ICET and BTT was 5.6 ± 0.3 sec (Table 3).

Next, we further classified 58 patients as having achalasia, EGJ outflow obstruction, distal esophagus spasm, hypercontractile esophagus, fragmented peristalsis, and ineffective esophageal motility and as normal according to HRM results. Of the three patients with distal esophageal spasm patients, 36.7% swallows showed ICET and two patients that had peristalsis breaks showed ICET. Of two patients with hypercontractile esophagus, one who had a disruption of peristalsis exhibited ICET. Seven patients demonstrated normal esophageal function and exhibited CET. Of the twelve patients of IEM, 70.4% swallows showed ICET, and all patients exhibited ICET; BTT was 8.9 ± 1.3 sec which was significantly longer than that in normal esophageal function patients (*P* < 0.05).

Interestingly, there were 7 patients who manifested hiatal hernia. Of these seven patients with hiatal hernia, 3 patients had fragmented peristalsis, 2 patients had IEM, and 2 patients with normal esophageal motility. 57.1% of the total 70 swallows showed ICET. According to the criterion that abnormal bolus transit was defined as ≥30% liquid swallows in individual, five patients showed ICET.

### 3.4. Correlation between Peristalsis Breaks and ICET

Bulsiewicz et al. [12] demonstrated that peristaltic contraction with breaks < 2 cm in the 20 mmHg isobaric contour or <3 cm in the 30 mmHg isobaric contour were associated with complete bolus clearance, and longer breaks predicted incomplete bolus clearance. In our study, we defined peristalsis breaks as ≥3 cm in the 30 mmHg isobaric contour. One EGJ obstruction, four hiatal hernia, two distal esophageal spasms, one hypercontractile esophagus, and six IEM patients that had peristalsis breaks exhibited ICET. 140 swallows of these patients were observed, and 102 swallows (72.9%) exhibited ICET (*r* = 0.73, *P* < 0.01).

## 4. Discussion

Dysphagia usually indicates impaired transport of a swallowed bolus through the esophagus [18]. Owing to traditional HRM could not give us information about bolus transit, and esophageal impedance is widely used to evaluate esophageal bolus transport. Therefore, HRIM was performed in 58 patients with NOD to analyze their characteristics and to preliminarily explore the pathophysiological mechanisms.

**Figure 2:**
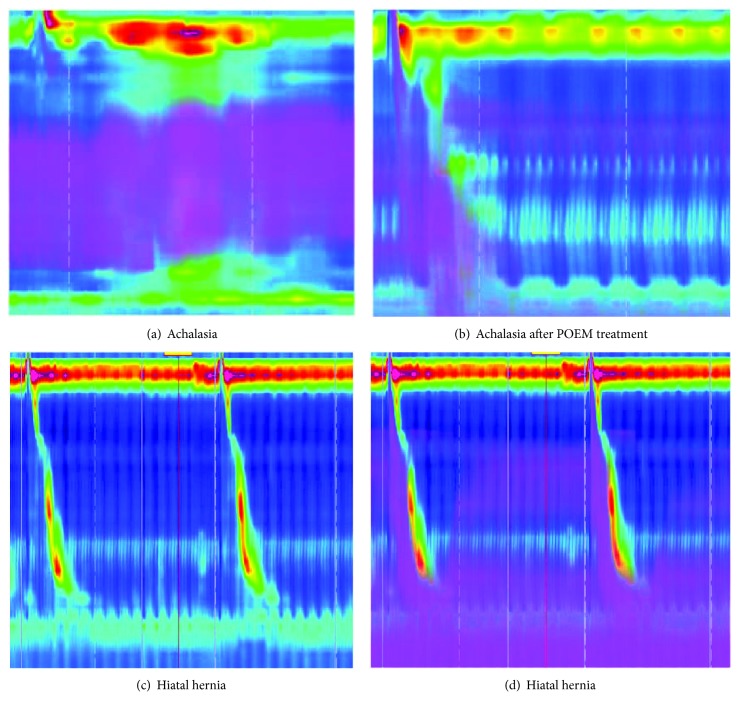
HRIM manifestation of one achalasia patient before and after peroral endoscopic myotomy (POEM) operation and one hiatal hernia patient. (a) One patient with II type achalasia in our study showed bolus retention in the esophagus body. (b) Four months after peroral endoscopic myotomy operation, the patient showed obvious reduced bolus retention and even exhibited proximal esophageal contraction. (c) One patient of hiatal hernia showed a 3 cm separation between the LES and CD during the baseline recording. (d) Gastroesophageal reflux occurred after the end of each peristaltic wave until the following swallow.

In our study, achalasia was found to be the most common cause of NOD, and HRIM results suggested that all achalasia patients manifest ICET irrespective of its type. Cho et al. [19] compared HRIM with timed barium esophagram (TBE) and demonstrated that there was excellent agreement between TBE and HRIM for assessing bolus retention at 5 min. Thus, HRIM may be used as a single test to assess bolus retention and motor function in the management of achalasia. Furthermore, Lin et al. demonstrated that bolus flow time was the only HRIM metric significantly associated with dysphagia questionnaire in achalasia patients [20]. One patient with II type achalasia in our study showed reduced bolus retention after performing peroral endoscopic myotomy and even exhibited proximal esophageal contraction (Figures 2(a) and 2(b)). Therefore, we suggest that HRIM could monitor the curative effect of achalasia, but it needs to expand sample size for further research.

EGJ outflow obstruction is defined by an elevated median IRP with some instances of intact or weak peristalsis such that the criteria of achalasia are not met. The pathophysiology of EGJ obstruction is not clear, and it presents a heterogeneous group with some individuals having an incomplete expression of achalasia and others likely having an undetected mechanical cause such as hiatal hernia or esophageal stenosis [21]. Previous study had shown that patients with EGJ outflow obstruction presented incomplete esophageal transit more frequently than normal controls [22], which was consistent with our results.

GERD is recognized to be a multifactorial disease, and its pathophysiology has not been fully clarified. We found the DCI values in patients with GERD were significantly lower than those in patients without GERD, which means impairment of esophagus clearance in GERD patients. Hiatal hernia is a known risk factor for GERD since it impairs the EGJ, leading to reduction in LESP and impairment of esophageal clearance. In our study, 5/7 hiatal hernia patients presented ICET. Recently, Torresan et al. [23] reported that a patient of hiatal hernia showed normal LESP and contractile integral and complete bolus clearance as well as absence of transient LES relaxation. However, after the end of each peristaltic wave, a gastroesophageal reflux was detected until the following swallow. The authors hypothesized that reflux is due to a transient increase in hernia sac pressure, when the hernia sac acts as a reservoir increasing its pressure to overcome the basal LES pressure, then the gastric content could reflux from the sac into the esophagus. In our study, we also found some patients of hiatal hernia showed a gastroesophageal reflux after the end of each peristaltic wave until the following swallow (Figures 2(c) and 2(d)). HRIM allowed a more accurate assessment and revealed a new mechanism through which hiatal hernia may lead to GERD.

**Figure 3:**
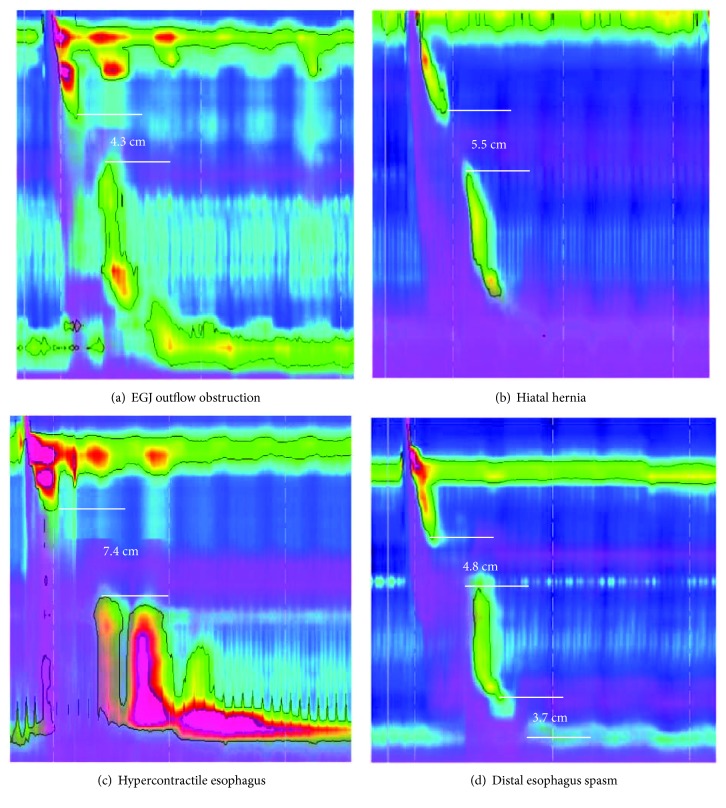
HRIM manifestation of peristalsis and incomplete esophageal transit (ICET). (a) One patient with EGJ outflow obstruction showed a 4.3 cm break in the 30 mmHg isobaric contour and exhibited ICET. (b) One patient of hiatal hernia had a 5.5 cm break in the 30 mmHg isobaric contour and presented ICET. (c) One patient of hypercontractile esophagus (presented hypercontractility of LES) showed a 7.4 cm break in the 30 mmHg isobaric contour and exhibited ICET. (d) One patient of distal esophagus spasm showed 4.8 and 3.7 cm breaks, respectively, and presented ICET.

HRIM depicts both esophageal pressure topography and bolus disposition on the same graphic, and thus the most comprehensive assessment of peristaltic integrity is achieved. Roman et al. [9] demonstrated that large (>5 cm) and small (2–5 cm) breaks in the 20 mmHg isobaric contour of the peristaltic contraction were associated with ICET for individual subjects. And Bulsiewicz et al. [12] also showed that breaks < 2 cm in the 20 mmHg isobaric contour or <3 cm in the 30 mmHg isobaric contour were associated with CET, and longer breaks predicted ICET. In addition, Almansa et al. [24] even found that chronic cough exhibited weak peristalsis with large breaks, and most of those patients exhibited poor bolus clearance of liquid swallows, which presented ICET. In our study, we found that 14 patients who had peristalsis break showed ICET, no matter whether it was EGJ obstruction, hiatal hernia, distal esophageal spasms, or hypercontractile esophagus (Figure 3). That means peristalsis breaks and ineffective swallows were the major factors associated with ICET, but distal esophagus spasm and hypercontractile esophagus may not.

The evaluation of bolus transit is crucial to the management and understanding of esophageal diseases. HRIM can provide a measure of bolus transit time (BTT) which could reflect bolus transport and clearance. Chen et al. [25] found that the upper limit of 95% of liquid and viscous bolus transit was <11.0 s. And Shi et al. [26] found that the median of BTT in normal Chinese population was 6.9 s. In our study, we found that the BTT in the patients with GERD was significantly longer than that in the patients without GERD, but the median values were less than 11.0 s. The role of BTT in evaluating the esophagus function needs further research.

However, a limitation of the present study is that the sample size of our study was limited, and this study was a retrospective analysis; thus, we did not have a follow-up data. It needs to expand sample size for further research. In addition, further investigation is required to identify the role of HRIM in evaluating the pathophysiological mechanisms and responding to treatment in NOD.

In summary, on the basis of our results, HRIM can be utilized to better determine the etiology of NOD and to accurately predict complete bolus clearance. It is also hopefully to shed light on the monitoring of achalasia and exploring the pathophysiological mechanisms of GERD.

## Figures and Tables

**Table 1:** Results of esophageal manometry in 58 NOD patients—LES and UES.

Esophageal motility	LESL (cm)	LESP (mmHg)	IRP (mmHg)	UESP (mmHg)	UESRP (mmHg)
NEMD					
Without GERD (*n* = 7)	2.9 ± 0.4	19.6 ± 5.6	6.2 ± 2.3	54.3 ± 5.7	7.8 ± 2.3
With GERD (*n* = 13)	2.9 ± 0.2	21.4 ± 6.1	7.2 ± 1.6	52.7 ± 6.1	9.1 ± 1.7
Achalasia (*n* = 28)	3.1 ± 0.1	36.8 ± 2.8	24.0 ± 2.0	54.9 ± 4.3	6.6 ± 0.8
EGJOO (*n* = 3)	3.4 ± 0.4	44.6 ± 13.4	33.4 ± 9.1	38.3 ± 0.6	3.8 ± 1.9
Normal (*n* = 7)	3.2 ± 0.2	19.4 ± 1.4	8.2 ± 0.7	61.0 ± 11.0	5.5 ± 1.9
*P* value	0.817	0.046	0.000	0.721	0.576

LESL: lower esophagus sphincter length; LESP: lower esophagus sphincter resting pressure; IRP: integrated relaxation pressure; UESP: upper esophagus sphincter resting pressure; UESRP: upper esophagus sphincter residual pressure; NEMD: nonspecific esophageal motility disorder; GERD: gastroesophageal reflux disease; EGJOO: esophagogastric junction outflow obstruction.

**Table 2:** Results of esophageal manometry in 58 NOD patients—esophagus body.

Esophageal motility	MP (mmHg)	DCI (mmHg·cm·s)	SC *n* (%)	IES *n* (%)	PB *n* (%)	Pan-EP *n* (%)
NEMD						
Without GERD (*n* = 7)	75.8 ± 10.5	1458.3 ± 216.8	1 (11.1)	1 (11.1)	4 (44.4)	0
With GERD (*n* = 13)	49.8 ± 5.8	699.1 ± 123.1	4 (30.8)	7 (53.8)	7 (53.8)	2 (15.4)
Achalasia (*n* = 28)	—	—	19 (67.9)	28 (100)	—	19 (67.9)
EGJOO (*n* = 3)	55.1 ± 7.2	1400.5 ± 428.2	2 (66.7)	1 (33.3)	1 (33.3)	0
Normal (*n* = 7)	128.4 ± 4.2	2344.6 ± 406.6	0	1 (33.3)	0	0
*P* value	0.000	0.002	0.000	0.000	0.000	0.000

MP: mean pressure; DCI: distal systolic integration; SC: synchronous contraction; IES: ineffective swallow; PB: peristalsis break; Pan-EP: panesophageal pressurize.

**Table 3:** Impedance results of esophageal manometry in 58 NOD patients.

Esophageal motility	*N*	ICET (%)	BTT (s)
NEMD			
Without GERD	7	49.1 ± 11.9	5.5 ± 0.3
With GERD	13	65.0 ± 11.6	8.1 ± 1.1
Achalasia	28	100.0 ± 0.0	—
EGJOO	3	90.0 ± 5.7	6.6 ± 1.2
Normal	7	3.5 ± 2.6	5.6 ± 0.3
*P* value	—	0.000	0.043

ICET: incomplete esophageal transit; BTT: bolus transit time.
